# A New Activated Sludge Model with Membrane Separation–Implications for Sewage and Textile Effluent

**DOI:** 10.3390/membranes11080589

**Published:** 2021-07-31

**Authors:** Derin Orhon, Ayse Begum Yucel, Guclu Insel, Seyda Duba, Tugba Olmez-Hanci, Bulent Solmaz, Seval Sözen

**Affiliations:** 1 The Science Academy, Istanbul 34349, Turkey; orhon@itu.edu.tr; 2 Environmental Engineering Department, Faculty of Civil Engineering, Istanbul Technical University, Maslak 34469, Istanbul, Turkey; aysebegumyucel@gmail.com (A.B.Y.); inselhay@itu.edu.tr (G.I.); seydaduba@gmail.com (S.D.); tolmez@itu.edu.tr (T.O.-H.); 3 Istanbul Water and Sewerage Administration, Istanbul 34060, Turkey; bsolmaz@iski.gov.tr

**Keywords:** MASM, new model for membrane activated sludge, particle size distribution, modified COD fractionation, captured COD fractions, denim processing wastewater

## Abstract

A new model for the activated sludge process with membrane separation is presented, based on the effective filtration size. A new size threshold is imposed by the membrane module. The model structure requires a modified fractionation of the chemical oxygen demand and includes chemical oxygen demand fractions entrapped in the reactor or in the flocs as model components. This way, it offers an accurate mechanistic interpretation of microbial mechanisms taking place in membrane activated sludge systems. Denim processing wastewater was selected for model implementation, which emphasized the significance of entrapped fractions of soluble hydrolysable and soluble inert chemical oxygen demand responsible for better effluent quality, while underlining the shortcomings of existing activated sludge models prescribed for systems with conventional gravity settling. The model also introduced particle size distribution analysis as a new experimental instrument complementing respirometric assessments, for an accurate description of chemical oxygen demand fractions with different biodegradation characteristics in related model evaluations.

## 1. Introduction

The activated sludge process has exhibited a remarkable display of major achievements since its invention more than a century ago. Based on studies devoted to exploring and understanding the different microbial mechanisms involved, heterotrophs and autotrophs with different metabolic functions could be harnessed and sustained as fractions of the same mixed culture, which paved the way for novel process configurations [1,2,3]. However, the process dragged along the only limping element, i.e., gravity settling, which is expected to perform the crucial function of separating the treated wastewater from the biomass to be recycled back into the biological reactor. Gravity settling often failed to function properly under the outburst of biomass, such as bulking and foaming. The low settling rate of flocculent sludge imposes restrictions on the biomass concentration in the reactor [4,5].

The membrane bioreactor (MBR) can be regarded as a groundbreaking milestone for the traditional activated sludge process; it was a major leap forward, which allowed the replacement of the space consuming problematic gravity settling with the membrane filtration of biomass. Aside from a high-quality effluent suitable for reuse, this innovation basically ended the restrictions of gravity settling on biomass concentration in the reactor, simply by uncoupling the floc morphology and the separation process resulting in a much smaller footprint. Supported by extensive research, the implementation of MBRs rapidly escalated, especially as a sustainable biological treatment system for municipal wastewater, mostly based on satisfactory practice [6,7,8].

The success in the evolution of the activated sludge process is largely due to the development of mathematical models enabling us to assess and evaluate the fate of substrate and biomass in the reactor. All major multicomponent activated sludge models (ASMs) are structured upon COD (Chemical Oxygen Demand) as the sole conserved parameter for organic carbon removal, which defines all components of substrate and biomass by means of COD fractionation based on different biodegradation characteristics [9]: ASM1 covered all the components and processes for the removal organic carbon and nitrogen; it was further expanded to ASM2 and ASM2d to include enhanced biological phosphorus removal. The following version, ASM3, was the first model to account for substrate storage [10]. It was soon modified into a model structure including simultaneous growth and storage (ASMGS), which provided a much better estimation of process behavior [11,12].

These models involve a single size threshold, often 450 nm, which is used to differentiate between particulate and “soluble” fractions [13]. This size range originates form the widespread use of 0.45 µm filters in wastewater characterization. This differentiation is important because: (i) it implies that particulate COD will be retained in the reactor as part of the biomass, whereas its soluble counterpart will not settle and will end up in the effluent when not biodegraded; (ii) it identifies a major COD fraction, i.e., *the soluble hydrolysable COD* between 450 nm all the way down to the range of readily biodegradable COD [13,14,15]. This model structure, while successfully implemented for conventional activated sludge systems with gravity settling, becomes different for MASs, i.e., *activated sludge configurations with membrane separation.* The reason for this statement is quite simple: for the membrane module, the 450 nm size threshold becomes obsolete but becomes equivalent to the effective filtration size of the selected membrane; this causes a drastic change in applicable COD fractionation, related mass balances and model structure. Therefore, the current practice of using ASMs for membrane systems might greatly distort and undervalue the system performance.

In short, activated sludge configurations with a membrane module, (MASMs), require a modified model structure for performance evaluation and control. In fact, reviews on the modelling of membrane bioreactor systems indicated that studies only involved the calibration of process kinetics and stoichiometry for existing ASMs, or modifying them for a better description of certain processes, such as the generation of soluble microbial products, (SMP). Constraints on particle size associated with the membrane module was not recognized as a significant issue for changing the model structure [16,17]. In this context, the main objective of this study is to define a new model, MASM, for the activated sludge process with a membrane module and evaluate its implications for system configurations operated at different sludge age (SRT) hydraulic retention time (HRT) levels configurations, based on a modified COD fractionation and particle size distribution applicable to textile wastewater selected for model implementation.

## 2. Conceptual Basis

The role of the membrane module on activated sludge modelling can best be recognized with a full insight of the relevant conceptual background, which essentially involves four major issues: First, *particle size distribution analysis* will be required as an additional experimental tool to better explore and utilize the complex nature of organic carbon (COD) in wastewater. Second, *the effective filtration size* of the membrane will be needed to determine a new size threshold for COD fractions in the model. It should be noted ASMs were originally based on respirometry but for MASs, physical characterization based on effective filtration size becomes more relevant and should support respirometry. Then, a *modified COD fractionation* based on the new size threshold will be introduced to define the necessary new model components. Finally, all these conceptual changes will lead to an *accurate mass balance* for assessing the fate of model components under selected operating conditions. 

### 2.1. Particle Size Distribution

**Figure 1:**
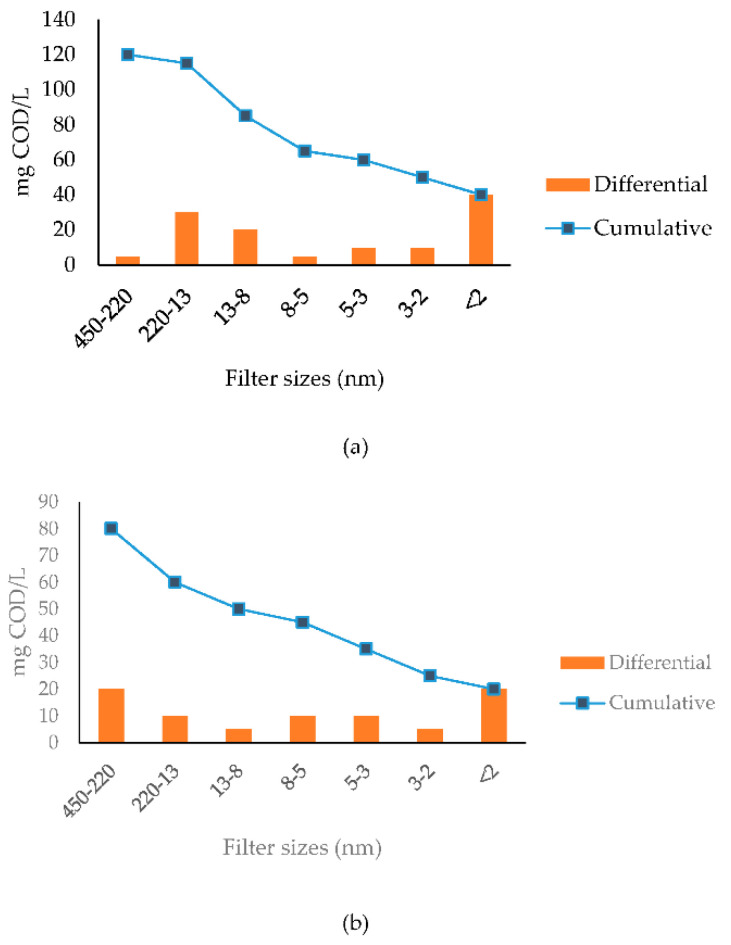
Related incremental and cumulative size distribution of soluble COD for (**a**) domestic sewage influent; (**b**) biological treatment effluent [29].

Obviously, the biodegradation of COD fractions relates to the particle size. This issue has been investigated since 1936, based on the limited information available at the time [18]. The concept of particle size was practically abandoned in the ASMs, which included only a single size threshold of 450 nm, which conveniently defined particular matter, i.e., either suspended or settleable solids. Recent research revived this concept: A procedure was developed for particle size distribution (PSD) analysis by means of sequential filtration/ultrafiltration from 1600 nm all the way down to 2 nm, providing a specific size fingerprint for selected parameters such as COD, total organic carbon (TOC), color, protein/carbohydrates, etc. [19,20,21]; so far, PSD analysis has found wide application for domestic sewage and different industrial wastewaters [22,23,24,25]. Lately, this method was implemented parallel to oxygen uptake rate measurements, mainly to complement COD fractionation for better interpreting the biodegradation characteristics [26,27,28]. Noyan et al. [29] provided a vivid example on coupling COD fractionation with particle size distribution, by matching PSD results with soluble COD fractions of domestic sludge. A similar coupling approach was also carried out on the effluent of biological treatment with a soluble COD escape of 80 mg/L, as shown in Figure 1.

Furthermore, inspection of the PSD data in Figure 1 revealed a significant aspect, which was not recognized before in published works, related to the fate of *S_H_* in biological treatment: The PSD analysis of the influent stream indicated a small fraction of 5 mg/L in the 220–450 nm size bracket. After biological treatment, *S_H_* was observed to increase to 20 mg/L in the same size range. This range is too high to be attributed to *S_P_* [30]. This finding can only be explained by the sequential hydrolysis of a fraction of slowly biodegradable COD, *X_S_* into *S_H_*, a very logical breakdown mechanism of particular organics to smaller size compounds. It should be noted that all ASMs stipulate that *X_S_* hydrolysis would yield directly readily biodegradable COD, *S_S_*. Therefore, the reported PSD analysis provided new and valuable information, which clearly shows that biological reactions not only generate residual metabolic products, but also soluble hydrolysable COD.

### 2.2. Effective Filtration Size

**Figure 2:**
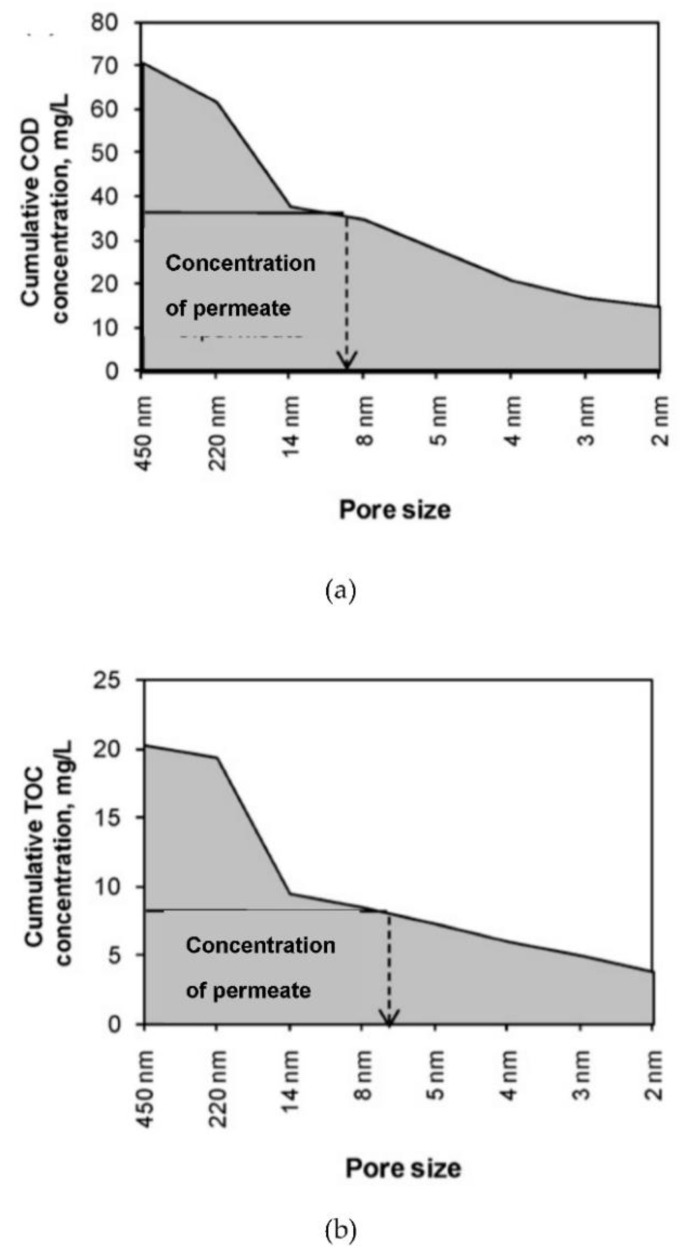
Cumulative size distribution for soluble COD indicating the effective filtration size [21].

In many studies investigating the behavior of extremely high rate MBRs fed with different substrates such as readily biodegradable mixture; acetate, starch, peptone mixture and sewage, observations always indicated that the soluble COD level in the reactor was significantly higher than that of the permeate [31]. Obviously, this implied the entrapment of a fraction of soluble COD, such as hydrocolloids, by the membrane module. These observations were further explored by a PSD analysis yielding a cumulative distribution of the reactor-soluble COD. As shown in Figure 2, the permeate COD marked on the size distribution profile indicates 8 nm as the effective filtration size of the membrane [21]. A number of similar experiments confirmed a range close to 8 nm (8–13 nm) for different parameters such as DOC, proteins and carbohydrates, regardless of the type of membrane module and the operation conditions adopted for the system [32,33,34]. The interesting feature of these findings was that the reported *effective filtration size* was always substantially lower than the nominal filtration size of the membrane module.

A much lower effective filtration size may easily be explained by the formation of a *cake layer* on the surface of the membrane, which fulfils the function of an additional separation barrier. This layer prevents a fraction of the pollutants from reaching the membrane surface, leading to the entrapment and accumulation of relatively large particles in the reactor volume [35,36]. While it would be recommendable to assess the effective filtration size for each case, there are sufficient grounds to suggest 8 nm as a default value for this parameter.

### 2.3. Modified COD Fractionation

Respirometry was the *magic touch* that basically created the current multicomponent activated sludge models [9]. Nowadays, the generation and model calibration of the oxygen uptake rate profiles (OUR) provide a specific fingerprint for the selected wastewater for identifying its COD fractions and the coefficients of the process kinetics involved [37]. Furthermore, they also serve to explore a wide spectrum of other interesting scientific issues such as the variability Monod equation with respect to sludge age [38]; the assessment of storage yield [39]; the inhibitory impact of xenobiotics on heterotrophic processes [40] and nitrifying cultures, etc. [41]. Respirometry receives additional support from direct experimental methods based on the assessment of inert COD fractions and residual metabolic products [42,43].

In the MAS configurations, the decline of the particle size threshold from the traditional level of 450 nm down to the *effective filtration size* puts an end to the “*particulate/soluble*” concept, which will only apply when differentiating slowly and rapidly hydrolysable COD fractions, *X_S_* and *S_H_*, because related process kinetics are well documented in the literature [44]. It induces significant changes on the structure of COD fractionation in the soluble (>450 nm) range: In this range, the soluble hydrolysable COD, *S_H_* is the major soluble COD fraction, covering a wide size range between 2–450 nm in wastewaters; it will be subdivided into two new COD fractions, *S_HC_*, which would be captured in the reactor and *S_H_*, smaller in size than the entrapment/capture threshold. Thus, the remaining part of *S_H_* after hydrolysis will bypass the membrane module with the effluent. Similarly, the soluble inert COD, *S_I_*, will be split into *S_IC_* and *S_I_*. The readily biodegradable COD, *S_S_* will not be affected by the effective filtration size because it is located at the far end of the size spectrum. This type of size fractionation will also be useful for fouling prediction.

## 3. Mass Balance and MASM Structure for Organic Carbon Removal

The backbone of the MASM was structured to adopt the modified COD fractionation, identifying all soluble COD fractions in the wastewater as different model components, with respect to the effective filtration size of the membrane module. This is a compulsory refinement for ASMs, for providing an accurate assessment of the fate and biodegradation of soluble COD and the soluble hydrolysable COD, *S_H_*, which often plays a key role in the quality of the process effluent. The settleable COD fraction, *X_SS_*, was also included in the model structure as a model component within the total biodegradable COD profile [45].

In this context, the structure of MASM essentially involved the basic template of all ASM models defined for organic carbon removal, previously implemented in many studies [46]. In this study, the adopted template was the one defining ASM1, modified for endogenous decay [47,48]; it basically included all soluble and particulate COD fractions—namely, *S_IC_*; *S_I_*; *S_SS_*; *S_HC_*; *S_H_*; *X_I_*; *X_SS_*; *X_S_*—in the wastewater as model components. Additionally, the soluble and particulate residual microbial products, *S_P_* and *X_P_*, were accounted for as part of endogenous respiration with the simplifying assumption of a decay-associated generation process [49]. Obviously, the model template also included the active heterotrophic biomass concentration, *X_H_*, and, finally, the dissolved oxygen concentration, *S_O_*, the basic parameter for the evaluation of the OUR profiles.

Based on the indication of particle size analysis [29], the adopted MASM structure stipulated that the breakdown of settleable COD would undergo a two-step mechanism, first hydrolysis to *S_HC_*—organics in the higher size range—and then from *S_HC_* to *S_S_*, as previously conceived.

Then, the fate of the overall soluble hydrolysable COD, *S_HT_* was accounted for as two different fractions, namely, *S_HC_*, the fraction that would be captured and returned to the reactor, and *S_H_*, the fraction that would bypass the membrane and exit the system with the effluent stream. This differentiation required totally different mass balance equations for the two fractions *S_HC_* and *S_H_*, when introduced into the MASM structure:

Equation (1) shows that *S_HC_* would be partly accumulated in the reactor the same way as biomass:

(1) $${\mathrm{QS}}_{\mathrm{HC}1}-P_{\mathrm{SHC}}\;-V\;(k_{h}\frac{S_{HC}/X_{H}}{K_{X}+S_{HC}/X_{H}}X_{H}-k_{hX}\frac{X_{SS}/X_{H}}{K_{XX}+X_{SS}/X_{H}}\;X_{H})=0$$

where

(2) $$P_{\mathrm{SHC}}=\frac{VS_{HC}}{{Ɵ}_{X}}$$

whereas Equation (3) defines the magnitude of *S_H_* that would escape the system with the effluent stream:

(3) $${\mathrm{QS}}_{H1}-{\mathrm{QS}}_{\mathrm{HE}}-V\;(k_{h}\frac{S_{H}/X_{H}}{K_{X}+S_{H}/X_{H}}\;X_{H})=0$$

These equations provide clear indication that *S_HC_* would be controlled by the sludge age (*SRT*), remaining and accumulating in the reactor, whereas *S_H_* would be basically controlled by the hydraulic retention time (HRT). A similar differentiation would be applicable and necessary for soluble residual COD fractions, *S_I_* and *S_P_*, in terms of the prediction of the system performance.

Obviously, the *X_SS_* component will not be considered in the modelling of the MAS configurations operated with a primary settler. Related rate expressions and basic stoichiometry were defined in a way compatible with ASM1 and presented in the usual matrix format in Table 1. Switching functions were omitted in the process rate expressions because *S_O_* and nutrients were supplied in excess in all model evaluations.

## 4. Implementation of MASM for Textile Wastewater

### 4.1. Evaluation Rationale

In this study, the proposed model was evaluated for textile wastewater, since textile processing involves many categories generating effluents with different characters [50]. A denim processing wastewater was selected mainly because: (i) data on COD fractionation and particle sized distribution are available in the literature; (ii) related biological treatment is mostly limited to COD removal; and (iii) it is a strong wastewater in terms of COD content, where the impact of modified COD fractionation and mass balance could be better visualized. The merit of the proposed model was evaluated by comparing the performance of the activated sludge with membrane separation with that of a traditional activated sludge system with gravity settling.

Obviously, the system behavior for the MAS operation was tested with the proposed model, MASM, while the parallel traditional activated sludge system was evaluated by means of ASM1, modified for endogenous decay. Model assessment of both systems was adjusted to visualize the impact of two key parameters, i.e., the sludge age, θ*_X_* and the hydraulic retention time, θ*_H_*; it was carried out for a set of different operating conditions. (i) In the first step, the modeling involved different footprints, i.e., different θ_*H*_ values for the two systems (mixed liquor suspended concentrations (MLSS) of around 10,000 mg/L for MASM and 4000 mg/L for ASM1 but the same θ*_X_*; this parameter was varied in different runs to designate the various applicable activated sludge configurations, i.e., super-fast; high rate; conventional, etc. (ii) In the second step, the models were implemented using the same θ*_H_* value, i.e., the same reactor volume and MLSS level (around 5000 mg/L) for the two systems.

**Figure 3:**
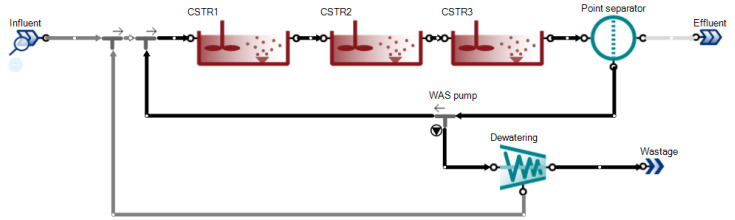
The SUMO configuration selected for MASM and ASM1.

The model evaluation utilized the SUMO simulation program; this software is specifically applicable for the modelling of activated sludge systems in municipal or industrial wastewater treatments. In this modelling study, the models were created by the SUMO program [51]. SUMO is a processor equipped with different multiple model options, where any number of configurations that could be created and related mathematical calculations are reflected with 100% accuracy. The configuration of this modelling consists of three CSTR that were linked to each other and at the end of the process a point separator acts as a membrane for MAS. From the point separator, the return activated sludge was linked to the first CSTR compartment and the waste activated sludge underwent a dewatering process. The flowrate of the system was defined as 10,000 m^3^/d where the volume and returned activated sludge (RAS) rate were changed for different runs. Figure 3 describes the configuration of the used model.

### 4.2. Selection of Wastewater Characteristics

It should be noted that the textile industry embraces various categories based on fabrics and processes involved, each generating a specific type of wastewater, quite different from one another. Related parameters for denim processing were selected from related experimental studies, which covered and reported both COD fractionation and particle size distribution, mainly because it was extremely important to provide accurate and reliable information concerning the fractions of soluble COD components that would be entrapped and captured in the reactor by means of membrane filtration. In this context, Table 2 defines modified COD fractionation and Table 3, the kinetic coefficients associated with denim processing effluents. Table 2 also includes characteristics of domestic wastewaters together with the textile wastewaters, mainly for the purpose of comparison.

## 5. Results

### 5.1. Fate of Soluble Hydrolysable COD

The characteristics of the textile wastewater in Table 2 reflect a clear indication that the soluble hydrolysable COD fraction, *S_HT_*, is the key COD component, affecting and controlling organic carbon removal. In fact, it corresponds to around 50% of the total soluble COD, *S_T_*, for the selected wastewater. The significance of *S_H_* may better be recognized in comparison with particulate COD, which will be retained in the reactor, and with the readily biodegradable COD, which will be fully utilized under all conditions.

### 5.2. Activated Sludge Configurations with Different HRT Levels

In this step, the design principles, and especially mixed liquor suspended solids (MLSS) recommendations and restrictions were observed both for the activated sludge systems with gravity settling and for membrane separation. Accordingly, MLSS levels were selected around 4000–4400 mg/L for traditional activated sludge configurations and 10,250–10,500 mg/L for the corresponding membrane separated systems. As expected, the selected design levels for MLSS yielded more than twice lower HRT values for MAS systems and, consequently, a much smaller footprint. Model evaluation was carried out with an SRT range of 1.0–8.0 d for characterizing “*superfast*”, high rate and conventional activated sludge configurations. The model outputs summarizing the fate of *S_H_* in the two systems are given in Table 4. Two observations in the results displayed require additional emphasis: (i) The effluent *S_HE_* levels were significantly lower for the MAS systems in the super-fast range operated at SRT levels below 2 d. In fact, model evaluation yielded a three-fold difference between *S_HE_* values at SRT of 1.0 d, and a two-fold difference when the SRT level was increased to 2.0 d. For the rest of the tested configurations, the *S_HE_* levels remained quite compatible, around 13–20 mg/L for high rate operations and below 10 mg/L for conventional treatments. (ii) The *S_H_* removal rates were always significantly lower for the ASM systems, despite the fact that it was evaluated as the total removal rate for activated sludge systems with gravity settling and the removal of the *S_H_* fraction, which could escape entrapment by the membrane. These observations deserve to be further discussed in the next section.

### 5.3. Activated Sludge Configurations Adjusted to Same HRT Levels

In this modelling step, the HRT and, consequently, the MLSS values were matched in parallel systems for each of the SRT levels tested. For super-fast system operation, the difference between the effluent *S_HE_* levels was further augmented; an *S_HE_* value of 18 mg/L should be compared with 95/L for the parallel operation at SRT of 2.0 d (Table 5). At an SRT of 1.0 d, the corresponding *S_HE_* values were 50 mg/L and 12 mg/L, respectively. For high rate and conventional operations, this approach yielded for MAS an almost complete removal of *S_H_* with effluent COD below the reliable detection limit (COD < 10 mg/L), always lower than their counterpart AS systems.

### 5.4. Effluent Quality

**Figure 4:**
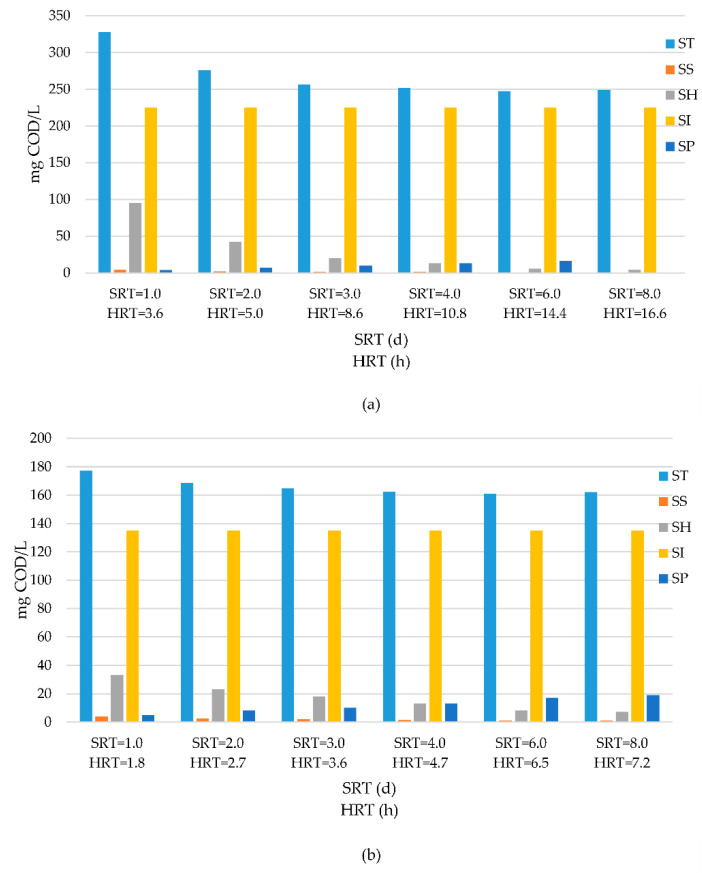
Effluent COD fractionation of textile wastewater operated at different HRTs in STEP 1 for (**a**) ASM1 and (**b**) MASM.

**Figure 5:**
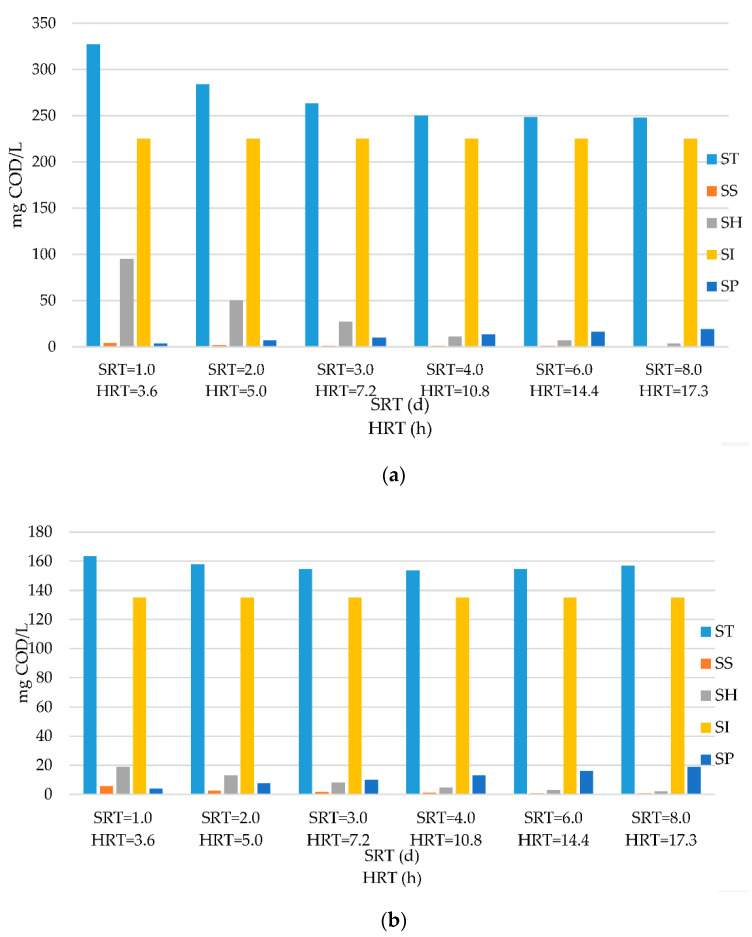
Effluent COD fractionation of textile wastewater operated at same HRTs in STEP 2 for (**a**) ASM1 and (**b**) MASM.

Model implementation was also extended to evaluate effluent quality in terms of all COD fractions involved. The modelling results were plotted in Figure 4 for the ASM1 and MASM applications considered with their design footprint (different HRTs) and Figure 5 when they were adjusted to the same HRT levels. The data displayed in these Figures are quite significant in the sense that they reflect the relative impact of different COD fractions on the effluent quality under different operation conditions; they underline the major role of residual COD fractions, especially the influent soluble inert COD, *S*_*I*1_, on the influent total soluble COD. Industrial wastewaters are usually characterized by high *S*_*I*1_ levels that threaten the effluent limitations. In fact, the *S*_*I*1_ values given in Table 2 were 225 mg/L for textile wastewater and only 18 mg/L for domestic sewage, different than 214 mg/L given for pre-treated tannery wastewater [26] and 3200 mg/L for leachate [22]. Therefore, it becomes crucially important to account for the captured fraction of *S*_*I*1_ in MAS systems to provide an accurate evaluation of system performance and effluent quality. This underlines the major shortcoming of the ASM models for evaluating and predicting the system performance of activated sludge configurations with membrane separation.

An inspection of the data in Figure 4 and Figure 5 clearly confirms the statements in the literature that SRT levels of 6–8 d for conventional activated sludge operations are merely selected to condition the biomass to obtain acceptable settling, because this range would secure almost the full removal of biodegradable COD, leaving behind residual COD fractions in the effluent. This is the exact image perceived, as the effluent COD, *S_TE_*, stabilized around 250–255 mg/L for the AS and 155–160 for MAS configurations. Slightly lower ranges were observed when the same HRT levels were selected for the two systems. The significant difference between effluent COD levels underlines the merit of membrane separation in MAS in capturing a portion of *S*_*H*1_ and preventing its discharge with the effluent. For lower SRT values, the magnitude of the soluble hydrolysable COD, *S*_*H*1_, becomes more pronounced with respect to residual COD components, causing a stepwise increasing divergence between the effluent quality of the ASM1 and MASM applications operated under the high rate and “*super-fast*” ranges. It should also be noted that effluent quality of activated sludge systems also suffers from biomass escape through gravity settling, which incorporates an additional particular COD load into the effluent. While the magnitude effluent particulate COD obviously depends upon the efficacy of operation and maintenance, it may be roughly estimated to vary between 30–60 mg COD/L.

## 6. Discussion

The structure of all ASM models effectively relies on a single size threshold of 450 nm to differentiate the soluble model components from the particulate ones. The COD fractionation comprised in this structure defines differences in the biodegradation characteristics with no concrete relation with particle size. It basically assumes that all remaining soluble components after biodegradation will be in the effluent stream and all particulate components will be kept in the reactor, except a very small fraction, which will escape gravity settling.

This picture is not compatible with AS systems coupled with membrane separation, and requires a novel modelling approach, where the conventional size threshold is drastically reduced to match the effective filtration size of the membrane. The available experimental information indicates the applicable new size threshold as 8–13 nm. This change implicates new model components among soluble COD fractions, specifically *S*_*HC*1_ and *S*_*IC*1_, larger-size portions of the hydrolysable COD, *S_H_*, and inert COD, *S_I_*, that would remain captured in the reactor. This is the basic novelty of the proposed model, MASM, which also underlines the basic shortcomings of the existing ASM when applied to membrane bioreactors.

This novel model structure provided by MASM is indispensable, mainly for two reasons: (i) *S*_*H*1_ is the major fraction of total soluble COD, *S*_*T*1_, in most wastewaters; in fact, it accounts for 48 to 52% of the *S*_*T*1_ level in the domestic sewage and textile wastewaters evaluated in this study. (ii) A high fraction of *S*_*H*1_ needs to be classified as *S*_*HC*1_, i.e., 76% for domestic sewage and 78% for textile wastewater; this fraction is quite significant and may be the cause of misinterpretations if not accounted for in the model structure. (iii) The same is true for *S*_*IC*1_, the entrapped fraction of the initial soluble inert COD, *S*_*I*1_, which becomes the decisive COD component in effluent quality, especially for the conventional operation range, which provides a nearly total removal of biodegradable COD.

Evaluation by MASM also underlines the significance of the selected reactor volume because the fate and removal of *S_H_* mainly depends on the selected HRT level. Therefore, a compromise should always be considered between better system performance and smaller footprint, especially for high rate MAS operations.

## 7. Conclusions

The newly proposed model for activated sludge configurations with membrane separation, MASM, involved a modified structure. The effective filtration size, a new size threshold imposed by the membrane module basically defines this modification. Accordingly, it defined a modified COD fractionation, accounting for entrapped COD fractions and including them as model components. This way, MASM provided an accurate mechanistic understanding and interpretation of microbial mechanisms taking place in *membrane activated sludge systems,* MASs.

MASM emphasized the significance of the captured portions of two COD fractions, namely, soluble hydrolysable COD, *S*_*H*1_, and soluble inert COD, *S*_*I*1_, which play a major function in the substantially better effluent quality associated with MAS systems.

MASM introduced the analysis of *particle size distribution* as a new experimental instrument and recommended it to be an integral complement of respirometry for an accurate description of COD fractions with different biodegradation characteristics in related model evaluations.

## Figures and Tables

**Table 1:** Matrix representation of the proposed model, MASM.

Model Components → Process ↓	*S_I_*	*S_IC_*	*X_I_*	*S_S_*	*S_H_*	*X_S_*	*S_O_*	*X_P_*	*S_P_*	*X_SS_*	*S_HC_*	*X_H_*	Process Rate
Growth of *X_H_*				$-\frac{1}{Y_{H}}$			$-\frac{1-Y_{H}}{Y_{H}}$					1	$\hat{\mu_{H}}\;\frac{S_{S}}{K_{S}+S_{S}}$ *X_H_*
Hydrolysis of *S_H_*				1	−1								$k_{hS}\frac{S_{H}/X_{H}}{K_{hS}+S_{H}/X_{H}}$ *X_H_*
Hydrolysis of *S_HC_*				1							−1		$k_{hS}\frac{S_{HC}/X_{H}}{K_{hS}+S_{HC}/X_{H}}$ *X_H_*
Hydrolysis of *X_S_*				1		−1							$k_{hX}\frac{X_{S}/X_{H}}{K_{hX}+X_{S}/X_{H}}$ *X_H_*
Hydrolysis of *X_SS_*										−1	1		$k_{hX}\frac{X_{SS}/X_{H}}{K_{hX}+X_{SS}/X_{H}}$ *X_H_*
Decay							−(1 − *f_S_* − *f_X_*)	f_X_	f_S_			−1	*b_H_X_H_*
Parameter	COD	COD	COD	COD	COD	COD	*S_O_*	COD	COD	COD	COD	Cell COD	

**Table 2:** Modified COD fractionation for textile and domestic wastewaters.

Parameters	Textile [29]	Domestic [20]
Total COD (mg/L), *C_T1_*	1340	415
Total soluble COD (mg/L), *S*_*T*1_	965	120
Total particulate COD (mg/L), *X*_*T*1_	375	295
Readily biodegradable COD (mg/L), *S*_*S*1_	280	40
Total soluble hydrolysable COD (mg/L), *S*_*HT*1_	460	62
Influent soluble hydrolysable COD (mg/L), *S*_*H*1_	100	15
Captured soluble hydrolysable COD (mg/L), *S*_*HC*1_	360	47
Total soluble inert COD (mg/L), *S*_*IT*1_	225	18
Influent soluble inert COD (mg/L), *S*_*I*1_	135	10
Captured soluble inert COD (mg/L), *S*_*IC*1_	90	8
Total particulate hydrolysable COD (mg/L), *X*_*ST*1_	360	253
Influent particulate hydrolysable COD (mg/L), *X*_*S*1_	162	113
Settleable biodegradable COD (mg/L), *X*_*SS*1_	198	140
Total particulate inert COD (mg/L), *X*_*IT*1_	15	42
Influent particulate inert COD (mg/L), *X*_*I*1_	7	19
Settleable inert COD (mg/L), *X*_*IS*1_	8	23

**Table 3:** Selected values of kinetic coefficients for textile wastewater.

	$\hat{\mu_{H}}$	*K_S_*	*b_H_*	*k_hS_*	*K_hS_*	*k_hX_*	*K_hX_*	References
								
	4.1	5	0.18	3	0.05	1	0.5	[52]
	5.3	5	0.14	3	0.05	1	0.2	[52]
	3.6	15	0.14	0.8	0.05	0.5	0.15	[52]
	-	-	-	2.5	0.4	0.1	0.5	[14]
	6	1	0.1	3.5	0.04	0.72	0.04	[53]
Selected for model	3.6	15	0.14	2.45	0.09	0.68	0.28	

**Table 4:** *S_H_* removal in ASM1 and MASM models operated for different footprints (different SRTs).

Textile Wastewater		ASM1			MASM		
	SRT (d)	HRT (h)	*S_HE_* (mg/L)	*S_H_* Removal (%)	HRT (h)	*S_HE_* (mg/L)	*S_H_* Removal (%)
							
Superfast	1	3.6	95	79	1.8	33	67
	2	5.8	42	91	2.7	23	77
							
High rate	3	8.6	20	96	3.6	18	82
	4	10.8	13	97	4.7	13	87
							
Conventional	6	14.4	5.5	99	6.5	8	92
	8	16.6	4	99	7.2	7	93

**Table 5:** *S_H_* removal in in ASM1 and MASM models operated at the same SRT values.

Textile Wastewater			ASM1		MASM	
	SRT (d)	HRT (h)	*S_HE_* (mg/L)	*S_H_* Removal (%)	*S_HE_* (mg/L)	*S_H_* Removal (%)
						
Superfast	1	3.6	95	79	18	82
	2	5	50	89	12	88
						
High rate	3	7.2	27	94	8	92
	4	10.8	11	98	5	95
Conventional						
	6	14.4	6.7	99	3.5	97
	8	17.3	3.6	99	3.2	97

## Data Availability

Not applicable.

## Nomenclature

AS	Activated sludge system
ASM	Activated sludge model
*b_H_*	Heterotrophic decay rate (1/d)
COD	Chemical oxygen demand (mg/L)
CSTR	Continuous stirred tank reactor
*f_S_*	Residual soluble metabolic fraction of endogenous biomass
*f_X_*	Residual particulate metabolic fraction of endogenous biomass
HRT	Hydrolic retention time (day)
*k_hs_*	Maximum hydrolysis rate for soluble COD (1/d)
*K_hS_*	Hydrolysis half saturation constant for soluble COD (g COD/g COD)
*k_hX_*	Maximum hydrolysis rate for particulate COD (1/d)
*K_hX_*	Hydrolysis half saturation constant for particulate COD (g COD/g COD)
*K_S_*	Half saturation constant for growth (g COD/g COD)
MAS	Activated sludge system with membrane separation
MASM	Model for activated sludge system with a membrane module
MBR	Membrane Bioreactor
$\hat{\mu_{H}}$	Maximum heterotrophic growth rate (1/d)
MLSS	Mixed liquor suspended solids (mg/L)
*P_SHC_*	Amount of soluble hydrolysable COD captured in sludge (kg COD/d)
*S_H_*	Soluble hydrolysable COD (mg/L)
*S_HC_*	Captured soluble hydrolysable COD (mg/L)
*S_HE_*	Soluble hydrolysable COD in the effluent (mg/L)
*S_I_*	Soluble inert COD (mg/L)
*S_IC_*	Captured soluble inert COD (mg/L)
*S_O_*	Dissolved oxygen concentration (mg/L)
*S_P_*	Soluble inert microbial product (mg COD/L)
SRT	Sludge retention time (d)
*S_S_*	Soluble readily biodegradable COD (mg/L)
WWTP	Wastewater treatment plant
*X_H_*	Active biomass concentration (mg COD/L)
*X_I_*	Particulate inert COD (mg/L)
*X_P_*	Particulate inert microbial product (mg/L)
*X_S_*	Slowly biodegredable COD (mg/L)
*X_SS_*	Settlable COD (mg/L)
